# Pomegranate (*Punica granatum* L.) Extract Effects on Inflammaging

**DOI:** 10.3390/molecules29174174

**Published:** 2024-09-03

**Authors:** Raffaele Cordiano, Luca Gammeri, Eleonora Di Salvo, Sebastiano Gangemi, Paola Lucia Minciullo

**Affiliations:** 1 Unit and School of Allergy and Clinical Immunology, Department of Clinical and Experimental Medicine, University of Messina, 98125 Messina, Italy; raffaelecordiano@gmail.com (R.C.); luca.gamm1993@gmail.com (L.G.); sebastiano.gangemi@unime.it (S.G.); 2 Department of Biomedical and Dental Science and Morphofunctional Imaging, University of Messina, 98125 Messina, Italy; eleonora.disalvo@unime.it

**Keywords:** aging, Alzheimer’s disease, central nervous system, inflammaging, inflammation, nutraceutical, oxidative stress, Parkinson’s disease, pomegranate, skin

## Abstract

Pomegranate is a notable source of nutrients, containing a considerable proportion of organic acids, polysaccharides, vitamins, fatty acids, and polyphenols such as flavonoids, phenolic acids, and tannins. It is also rich in nutritionally important minerals and chemical elements such as K, P, Na, Ca, Mg, and N. The presence of several bioactive compounds and metabolites in pomegranate has led to its incorporation into the functional food category, where it is used for its numerous therapeutic properties. Pomegranate’s bioactive compounds have shown antioxidant, anti-inflammatory, and anticancer effects. Aging is a process characterized by the chronic accumulation of damages, progressively compromising cells, tissues, and organs over time. Inflammaging is a chronic, subclinical, low-grade inflammation that occurs during the aging process and is linked to many age-related diseases. This review aims to summarize and discuss the evidence of the benefits of pomegranate extract and its compounds to slow the aging processes by intervening in the mechanisms underlying inflammaging. These studies mainly concern neurodegenerative and skin diseases, while studies in other fields of application need to be more practical. Furthermore, no human studies have demonstrated the anti-inflammaging effects of pomegranate. In the future, supplementation with pomegranate extracts, polyphenols, or urolithins could represent a valuable low-risk complementary therapy for patients with difficult-to-manage diseases, as well as a valid therapeutic alternative for the topical or systemic treatment of skin pathologies.

## 1. Introduction

The pomegranate (*Punica granatum* L.) is a member of the Punicaceae family, characterized by the presence of red flowers and large, sweet fruits. Moreover, the fruit is delimited by a membrane, the pericarp (peel), which contains the arils (the external part of the seed). Each seed is encased in a sac containing transparent juice. Thin membranes extend from the pericarp into the fruit, forming a latticework in which the arils are suspended.

The pericarp constitutes almost half of the weight of the fresh fruit; the seeds represent 10% of the weight, and the arils 40% [1].

The pomegranate originated in the Middle East, from where it was widely distributed throughout the Mediterranean region, later spreading east and west to China, India, Mexico, California, and the American Southwest [2]. This fruit exhibits a distinctive irregular round shape with a coriaceous peel. The fruit itself displays a spectrum of colors, from yellow to green or from pink to red, depending on the variety and phase of ripening. Furthermore, the fruit is classified as a false berry and may be consumed fresh or utilized in the manufacture of jams and juices, as well as in the production of oil, wine, and extract supplements.

The popularity of the fruit is increasing among growers and consumers worldwide. In 2017, the global production of pomegranate was approximately 3.8 million tons [3]. Various factors contribute to this expansion, including the fruit’s tolerance to a range of biotic and abiotic stresses, high yield potential, improved fruit quality, and higher nutraceutical value. The relatively low maintenance costs associated with pomegranate cultivation also contribute to its growing popularity [4].

The pomegranate fruit is a notable source of nutrients, including dietary fiber, polysaccharides, vitamins, fatty acids, and polyphenols [5,6]. In fact, pomegranate is rich in minerals such as sodium (3 mg/100 g), which is important for cellular homeostasis and helpful in maintaining physiology; potassium (236 mg/100 g), responsible for fluid balance; calcium (10 mg/100 g) with its numerous functions in the human body as a main constituent of bones and teeth; and magnesium (12 mg/100 g), helping in maintaining a constant metabolism and healthy bones as well. Moreover, the fruit contains protein (1.67 g/100 g) and fat (1.17 g/100 g) (information available at https://fdc.nal.usda.gov/fdc-app.html#/food-details/169134/nutrients, (accessed on 15 July 2024).

The main bioactive compounds in pomegranate are polyphenols such as tannins (punicalagin and other ellagitannins), flavonoids (flavonols, proanthocyanidins, and anthocyanidins), and phenolic acids (gallic, ellagic, caffeic, ferulic, and cinnamic acids). The content of tannins is between 193 and 420 mg/g of dry matter in pomegranate peel, while flavonoids account for between 84 and 134 mg [3]. The phenolic acid content depends on the geographical environment where the pomegranates are planted. In Tunisian pomegranate peels, the content of gallic acid, ellagic acid, and caffeic acid are 123.79, 35.89, and 20.56 mg/100 g, respectively [7].

Pomegranate seeds contain 7–27% oil, which is a rich source of polyunsaturated fatty acids, including linolenic and linoleic acids, as well as other lipids, such as oleic, stearic, and palmitic acids [8]. The arils contain mainly water (85%), sugars (10%), and pectin (1.5%) but are also a source of phenols, flavonoids, and anthocyanins [1].

The presence of several bioactive compounds, mainly polyphenols, in pomegranate has led to its incorporation into the functional food category, where it is used for its numerous therapeutic properties. These include antiviral, bactericidal, antifungal, immune modulation, stomachic, laxative, styptic, diuretic, and anthelmintic properties [9].

In light of these findings, several studies have investigated the role of pomegranate bioactive compounds in different diseases, including aging- and inflammation-mediated conditions [3,10,11,12,13].

Aging is a process characterized by the chronic accumulation of damage, progressively compromising cells, tissues, and organs over time. Depending on the underlying causes, aging can be classified as intrinsic (chronological) or extrinsic [14]. Various factors influence this process, such as genetic predisposition, epigenetic modifications, and environmental factors. Extrinsic aging, also known as photoaging, is mediated by exposure to external factors such as UV radiation, air pollution, smoking, temperature changes, as well as viruses, bacteria, and parasite infections. Intrinsic aging occurs as a consequence of physiological processes and has genetically determined characteristics, though epigenetic and post-translational mechanisms are considered crucial pathways [15]. However, it is difficult to separate these two aspects of aging, as they synergistically contribute to cumulative deleterious damages, leading to the onset of diseases [16].

The aging process is dynamic and characterized by continuous remodeling. Indeed, it is also an adaptive process, as over the years, the immune system gradually adapts to exposure to harmful internal and external agents, modifying the body’s microenvironment [17]. A key role in this remodeling theory is played by DNA repair, apoptosis, immune response, oxidative stress, and inflammation. These anti-aging mechanisms operate at different levels: molecular (i.e., DNA repair), cellular (i.e., apoptosis, autophagy, and cell senescence), systemic (anti-inflammatory response), and even at the organismic level (i.e., behavioral response to stressors). Therefore, counteracting age-related damage requires all these mechanisms to interact effectively with each other [18].

Among the aging theories, one called ‘‘inflammaging’’ is based on the activation of chronic subclinical low-grade inflammation that occurs during the aging process [19]. The NF-κB signaling complex represents innate immunity’s primary regulator and seems responsible for the inflammaging process [20].

The role of reactive oxygen species (ROS) and oxidative stress is fundamental to the phenomenon of inflammaging. Inflammation leads to an increase in ROS levels, which in turn is a key component in the maintenance of chronic inflammation, activating important pathways in the pathogenesis of age-related diseases [21].

Chronic low-grade inflammation also plays a role in skin aging and the genesis of dermatological pathologies, although few studies are available [22,23]. Therefore, at the basis of aging, there is a precarious balance between the inflammatory state and the anti-inflammatory response. In fact, when inflammation takes over the body’s defenses, ‘unsuccessful’ aging occurs, leading to a rapid onset of inflammatory and age-related disorders, as well as a short life expectancy. On the contrary, the capacity for a strong anti-inflammatory response is the key to longevity [18,24]. Therefore, it seems appropriate to promote one’s anti-inflammatory activity to maintain good health during the aging process. This means that a combination of a healthy lifestyle with an adequate diet is essential for improving aging.

Pomegranate and its products have long been used in traditional medicine for their antioxidant and antimicrobial activities. Today, its bioactive compounds, mainly polyphenols, are used in metabolic [25,26], cardiovascular [27,28], and oncological diseases [29,30]. In fact, the benefits of polyphenols have been investigated by various authors [31,32,33,34], focusing also on their anti-inflammatory and antioxidative stress effects [10,35,36].

Antioxidants, such as polyphenolic compounds from secondary plant metabolism, can stabilize free radicals and mitigate their harmful effects. In particular, ellagic acid is believed to be a key contributor to pomegranate’s antioxidant power [37].

All the studies cited above focus on the pomegranate’s antioxidant or anti-inflammatory properties or its beneficial effects in a single field of application (dermatological, metabolic, cardiovascular, and neurological). Despite that, no work has focused on the more complex concept of inflammation, and no author has evaluated how pomegranate can slow down this phenomenon across different organs and systems. This review aims to collect all the knowledge reported in the literature regarding the benefits of pomegranate and its metabolites, mainly polyphenols, in slowing down aging processes by intervening in the mechanisms underlying inflammaging.

## 2. Materials and Methods

We conducted a PubMed search using the following keywords: “pomegranate”, “ag-ing”, and “inflammaging”. Our analysis included all research articles in English and conducted in vivo or in vivo, without a time limitation, exploring the potential benefits of pomegranate extracts in age- and inflammation-related conditions.

Our search yielded 110 articles. We excluded reviews, systematic reviews, and meta-analyses. After eliminating duplicate articles, we identified 63 eligible articles. Of these, 26 concerned the use of pomegranate or its metabolites in the treatment of neurodegenerative diseases (18 on Alzheimer’s disease and 8 on Parkinson’s disease), 32 concerned skin-aging and skin inflammatory processes, 2 concerned protective effects on the cardiovascular system, 2 on metabolic diseases, and 1 on age-related infertility.

## 3. Results

### 3.1. Pomegranate and Central Nervous System

Aging plays a role in the genesis of neurodegenerative diseases. Inflammaging and immunosenescence can modulate the activity and reactivity of neuronal immune cells, leading to a low-grade chronic inflammation defined as neuro-inflammaging [38]. Some authors have demonstrated that a progressive loss of immune system functionality is essential in the pathogenesis of Alzheimer’s disease (AD) [39]. In these patients, aging, microglial dysfunction, and an inadequate peripheral immune response lead to the onset of neurodegenerative processes. Also, in Parkinson’s disease (PD), the interaction between inflammaging and immunosenescence predisposes to neurodegenerative processes [38].

To summarize, three mechanisms underlie neurodegenerative diseases: inflammation, oxidative stress, and the deposit of misfolded proteins.

The efficacy of pomegranate and its components against neurodegenerative diseases is widely demonstrated, as reported in the recent review by Emami Kazemabad et al. [40]. A long-term supplementation diet with pomegranate has proven effective in animal models in reducing neuroinflammation and improving visual memory [41].

#### 3.1.1. Pomegranate in Alzheimer’s Disease

AD is the most widespread form of dementia and is a progressive neurodegenerative disease characterized by deficits in mnemonic functions, language, and thinking skills [42]. The majority of patients present with a late-onset form of the disease (LOAD), which is sporadic. From a pathogenetic point of view, the disease is characterized by the cerebral deposition of β-amyloid substance in the form of plaques (Aβ plaques) and neurofibrillary tangles composed of the microtubule-associated τ protein [43]. These deposits are responsible for the loss of synapses between neurons and their death, with a progressive loss of brain tissue.

The deposition of these substances determines the activation of microglia, favoring the continuous release of pro-inflammatory cytokines from the IL-1β family. Furthermore, in AD, dysregulation of the GABAergic mechanism is created, and the inhibitory role of GABA on activated microglia is, therefore, lost, favoring an inflammatory microenvironment [44].

The effectiveness of treatment with inhibitors of acetylcholinesterase (AChE inhibitors), an enzyme implicated in the degradation of acetylcholine, confirms the protective role of cholinergic neurons [45].

Another element implicated in neuronal damage is oxidative stress. The production of ROS is increased in the brains of subjects with AD, leading to an increase in oxidative damage to the tissues. The increase in the production of free radicals is a consequence of aging processes, which also determines dysfunction in antioxidant action mechanisms [46].

Aging phenomena underlie the pathology and represent the main risk factor for late-onset Alzheimer’s disease.

Pomegranate and its compounds have demonstrated effectiveness in reducing risk factors for the development of AD and serve as nutraceutical support to slow down the progression of the disease due to their neuroprotective effects [47].

In the literature, there are several studies, both in vitro and on animal models.

Choi et al. [48] studied the pomegranate extract’s antioxidant and neuronal protective effects on PC12 cells. Ethanol extracts of the fruit showed a protective effect on PC12 cells against hydrogen peroxide-induced oxidative stress, decreasing neuronal death caused by Aβ and oxidative stress. Studies on mouse models have instead demonstrated that pomegranate can reduce learning and memory deficits induced by Aβ.

Velagapudi et al. [49] demonstrated the anti-neuroinflammatory effects of pomegranate juice and its extracts in vitro. Pomegranate juice was analyzed using high-performance liquid chromatography and standardized to ellagitannins, such as punicalagin (80–85%) and free ellagic acid (1.3%). Through an enzymatic immunoassay, the authors demonstrated pomegranate’s dose-dependent reduction in COX-2-dependent PGE2 production in IL-1β-stimulated SK-N-SH cells.

The first evidence of the anti-inflammatory effects of pomegranate juice in animal models dates back to 2015. Essa et al. [50] evaluated the anti-inflammatory effects of long-term pomegranate supplementation in APPsw/Tg2576 mice models. Supplementation reduced pro-inflammatory cytokines (IL-1β, IL-6, and TNF-α) and inhibited eotaxin activity.

In vivo evidence of the antioxidant effects of pomegranate extracts was described in 2008, yet by Kumar et al. [51]. Vitamin C and pomegranate extract supplementation for 21 days decreased lipid peroxidation and increased antioxidant glutathione levels in brain tissue of aged and scopolamine-treated young mice.

Subash et al. [52] evaluated the antioxidant effects of pomegranate on AD transgenic mouse models. Supplementation with pomegranate (4% of the diet) for 15 months induced a reduction in lipid peroxidation and restored the activity of antioxidant enzymes such as superoxide dismutase, catalase, glutathione peroxidase, glutathione, and glutathione S-transferase. Furthermore, the activity of the sodium–potassium pump and AChE was restored, which was instead altered in control mice.

Pomegranates slow down the formation of senile plaques.

The anti-amyloidogenic activity of pomegranate was studied in animal models in which an alteration in the levels and ratio of Aβ42 to Aβ40 proteins was observed after a three-week supplementation [53].

Braidy and his group [54] confirmed the neuroprotective effects of pomegranate supplementation and its ability to improve synaptic activity in mouse models of AD. Long-term supplementation reduced the loss of synaptic proteins like PSD-95, Munc18-1, and SNAP25. Furthermore, the integration diet ameliorated the phosphorylation of calcium/calmodulin-dependent protein Kinase IIα and Cyclic AMP-Response Element Binding Protein, which enhanced autophagy and activated the phophoinositide-3-kinase-Akt-mammalian target of the rapamycin signaling pathway. These events led to a reduction of the β-site cleavage of Amyloid Precursor Protein [54].

In the same year, Cardeira Morzelle et al. [55] studied differences between mice chronically infused with Aβ1–42, treated or not treated with pomegranate peel extract. They observed a reduction in amyloid plaque density, acetylcholinesterase enzyme activity, and lipid peroxidation in mice treated with pomegranate. Therefore, a reduction in TNF-α concentration was also observed with an improved expression of the neurotrophin BDNF.

In light of this beneficial effect, the potential benefit of pomegranate extracts administered through nanoparticles was evaluated. Studies on an AlCl 3-induced AD rat model treated with nanoparticles loaded with a 0.68% pomegranate extract showed an enhancement in the efficacy of alleviating oxidative stress, lipid peroxidation, and histopathological hallmarks in AD rat brains [56].

Several authors have tried to characterize the compounds in pomegranate with greater pharmacological activity and their mechanisms of action.

Khokar et al. [57] subjected pomegranate peels extracted with chloroform, ethyl acetate, and butanol to phytochemical screening. They subsequently quantified the total content of phenols and flavonoids and studied the neuroprotective potential by evaluating antioxidant activity and the inhibition capacity of acetylcholinesterase (AChE). These in vivo studies demonstrated the highest content of antioxidant substances in the butanol extract, revealing promising neuroprotective activity [57].

A more recent study demonstrated the powerful inhibitory activity of ethanol extracts of pomegranate peel and seed compared to water extracts. This extraction method was not evaluated in the previously cited study. The AChE inhibitory activity is related to the different content of bioactive metabolites in the different extracts. The mainly represented compounds were ellagic acid, catechin, epigallocatechin gallate, epicatechin, nicotiflorin, astragalin, gallic acid, epigallocatechin, quinic acid, tannic acid, aconitic acid, hesperidin, isoquercitrin, rutin, fumaric acid, cosmosin, luteolin, and epicatechin gallate [58].

AChE and β-secretases (BACE1) inhibition represent primary mechanisms through which polyphenols exert their anti-AD activity. In 2022, a study revealed the powerful activity of gallagic acid and castalagin in inhibiting these enzymes. Both compounds reduced the secretion of Aβ peptides in vitro, significantly reducing the expression of BACE1 and APPsβ without affecting APP levels. Furthermore, co-incubation studies of Aβ42 with gallagic acid demonstrated how the compound is capable of reducing the production of ROS induced by Aβ42 [59].

Rojanathammanee et al. [60] identified ellagic acid and punicalagin as highly active in inhibiting neuroinflammation. The authors demonstrated in mouse models the effect of these polyphenols in reducing the activity of nuclear factor of activated T-cells and Aβ-stimulated TNF-α secretion, reducing microgliosis and slowing the progression of the disease.

A study on mouse models demonstrated how intraperitoneal injection of LPS resulted in memory impairment and how repeated injections of LPS caused an accumulation of Aβ1–42 in the hippocampus and cerebral cortex due to the induction of activity of beta- and gamma-secretases and increased expression of APP. The improvement induced by three weeks of pretreatment with sulindac sulfide, a suppressive agent of LPS-induced amyloidogenesis, served as a counterproof of the observation [61]. In 2017, the same group demonstrated the ability of punicalagin to inhibit LPS-induced memory impairment. In their in vivo studies, the authors observed a reduction in LPS-induced expression of iNOS and COX-2. Moreover, they observed a reduction in ROS, NO, TNF-α, IL-1β, and down-regulation of APP and BACE1 expression [62].

The efficacy of punicalagin was recently confirmed in an in vivo aging mouse model induced by D-galactose [63]. Supplementation with punicalagin inhibited microglial activation and astrocytosis, reducing neuroinflammation and improving learning and memory deficits. Furthermore, punicalagin reduced NLRP3 inflammasome activation, as well as levels of pro-inflammatory cytokines, ROS, and MDA [63].

Punicalagin has also shown significant effects on antioxidant activity. Indeed, pretreatment of human neuroblastoma IMR-32 cells with punicalagin at a dose of 20 μM increased the enzymatic activity of Methionine sulfoxide reductase A (MsrA). In this study, punicalagin was superior to resveratrol, a phenol with high antioxidant activity, at the same dose [64].

A reduction in MsrA activity leads to changes in Aβ solubility properties and induces mitochondrial dysfunction, favoring the neurodegenerative process [65].

Digestive and intestinal absorption are essential in the metabolism of these compounds and, therefore, the role of the microbiome. Consequentially, the intestine–brain axis also has a relevant role. According to the review by Gates et al. [66], pomegranate regulates gut microbiota and could have a protective effect on the CNS, regulating neuroinflammation. Some in vivo studies have shown that pomegranate regulates the metabolism of intestinal microbiota by increasing the release of gamma-aminobutyric acid (GABA) and short-chain fatty acids [67,68]. Indeed, GABA is inhibited in AD, and short-chain fatty acids have anti-inflammatory properties [68].

Some authors have hypothesized the possibility of incorporating polyphenols into polymeric nanoparticles (based on polyethylene glycol, polylactic acid, or polyethyleneimine) to make them more easily permeable across the blood–brain barrier [69]. The clinical success of the drug GV-971, an algae derivative that modulates the gut–brain axis, recently highlighted the importance of this axis in disease treatment [66].

Ellagitannins in pomegranate are not absorbed as they are or found in circulation. These are metabolized at the intestinal level and absorbed as urolithins.

Espin et al. [70] demonstrated the metabolism of ellagitannins for the first time in 2007. The intestinal microbiota metabolizes ellagic acid, producing several compounds: tetrahydroxy- (urolithin D), trihydroxy- (urolithin C), dihydroxy- (urolithin A), and monohydroxy- (urolithin B metabolites) dibenzopyran-6-one. These urolithins are, therefore, absorbed in relation to their increasing lipophilicity [70].

In 2016, some computational studies evaluated the ability of compounds extracted from pomegranate to cross the blood–brain barrier. Urolithin A exhibits anti-aging properties among the different metabolites of urolithin. Furthermore, urolithins are effective in preventing β-amyloid fibrillation in vivo, and methyl-urolithin B has demonstrated neuroprotective effects in studies using *Caenorhabditis elegans* [71,72].

DaSilva et al. [73] studied the effects of urolithins at a dose of 10 μM on LPS-stimulated BV-2 microglia. Subsequently, the effects of pomegranate extracts on the expression of inflammatory genes in the hippocampal tissue of AD transgenic mice were evaluated. The authors demonstrated that urolithins reduced levels of nitric oxide, IL-6, PGE2, and TNF-alpha in LPS-stimulated BV-2 microglia. This reduced apoptosis and levels of caspase 3/7 and 9 induced by oxidative stress. However, the differences in the expression of biomarkers of inflammation in the hippocampus between mice treated with high-dose extracts and controls were not statistically significant [73].

Urolithin A is also a potent inducer of mitophagy (effect demonstrated by both in vivo and in vivo studies) [74].

Mitochondrial dysfunction is responsible for the neurodegenerative processes implicated in AD. It results from increased oxidative damage to mtDNA and reduced electron transport chain (ETC) functionality [75]. Furthermore, the dysfunction of the mitophagy process is implicated in aging processes and neurodegenerative diseases. In fact, mitophagy represents a mechanism of protection against oxidative stress, favoring mitochondrial homeostasis, and targeting mitophagy to slow neuronal damage [76]. Moreover, urolithins inhibit the activity of BACE1, leading to a reduction in amyloid beta fibrillation [72].

Table 1 summarizes the characteristics and main results of the studies discussed above.

#### 3.1.2. Pomegranate in Parkinson’s Disease

Studies in the literature regarding using pomegranate extracts to prevent or treat PD are limited. The disease is characterized by the loss of dopaminergic neurons in the substantia nigra pars compacta located in the midbrain. This condition is associated with the intraneuronal accumulation of Lewy bodies, which are cytoplasmic inclusions consisting of insoluble aggregates of alpha-synuclein [77]. Furthermore, the degradation of dopamine by monoamine oxidase B (MAO-B) leads to the accumulation of glutamate and excessive oxidative stress with the release of free radicals, which causes excitotoxicity [78]. It seems that environmental factors (toxins, drug abuse, and stress) and aging can promote the chronic low-level inflammation that we identify in neuro-inflammaging [79].

The first study on the efficacy of pomegranate in PD was conducted in vitro in 2013. The authors evaluated the effects of four pomegranate varieties on in vitro models (MPTP-induced neurotoxicity). They studied the activity of extracellular LDH, intracellular levels of NAD(+) and ATP, and levels of endogenous antioxidants (lipid peroxidation products, catalase activity, SOD, and glutathione peroxidase). In particular, two varieties, Helow and Malasi, were shown to determine a reduction in MPTP-induced neurotoxicity by attenuating alterations in redox function markers, showing how it could be helpful to select pomegranate varieties [80]. This could be determined by the higher gallic acid equivalents in these two varieties.

Tapias and his team [81] obtained conflicting results in 2014. They evaluated the effects of pomegranate juice on a rotenone-induced PD model in rats. Contrary to what was expected, the histopathology evaluation showed increased nigrostriatal terminal depletion and dopamine neuron loss. The authors hypothesized that polyphenols might have pro-oxidant effects under several conditions, increasing NO-mediated stress, iNOS, and NF-κB expression and inducing caspase-3 activation. The authors also hypothesized that the detrimental effects of pomegranate juice in these mouse models could be related to the use of rotenone to induce pathology in these animal models. The antioxidant response may be diminished when there is substantial ongoing oxidative stress, as in the rotenone model, and pomegranate juice may exhibit these pro-oxidant effects.

However, subsequent studies provided contrary evidence on rotenone-induced models, confirming the potential beneficial effect of pomegranate juice.

In 2019, another study on a rotenone-induced rat model of PD provided new evidence of the protective role of pomegranate-derived compounds [82]. In particular, they observed the effects of ellagitannins-derived metabolites on the postural stability of rat models, brain neurodegeneration, and oxidative stress. The results showed improved stability and reduced neuronal damage, with enhanced protection against oxidative stress and α-synuclein aggregation. Furthermore, the authors evidenced the distribution of urolithin A in the brains of treated rats [82].

Subsequently, the same authors evaluated the impacts of pomegranate juice on motoric, olfactory, and neurochemical alteration [83]. Pomegranate juice supplement has led to an increase in vertical activity and olfactory function in rats treated with rotenone, enhancing the release of dopamine and 3,4-dihydroxyphenylacetic acid (DOPAC) in the midbrain of rats [83].

A recent study by Fathy et al. [84] supports evidence of the neuroprotective effects of pomegranate juice and seed extract in PD models. Indeed, juice and seed extracts exhibited similar protective effects against paraquat-induced neurotoxicity in mice. Pretreatment with extracts of pomegranate juice or its seed extract increased the levels of tyrosine hydroxylase (TH), striatal dopamine, and DOPAC in the striatum while also reducing oxidative stress. A further benefit is represented by the ability to significantly reduce the expression of the NF-κB gene, as well as apoptosis phenomena and levels of pro-inflammatory cytokines, CD11b, and TGF-β. Finally, the authors observed a significant increase in the levels of anti-inflammatory cytokines such as IL-10, glial cell-derived neurotrophic factor (GDNF), and ATP [84].

Among the various compounds contained in the juice, ellagic acid has demonstrated its effectiveness in studies by Sarkaki [85] and Baluchnejadmojarad [86].

Ellagic acid supplementation in PD models of rats induced with 6-hydroxydopamine (6-OHDA) restored the antioxidant systems, exercising neuroprotective effects. The group treated with ellagic acid at 50 mg/kg/2 mL per die obtained an improvement in motor activity and electrophysiological performance as registered by EEG. Moreover, it was recorded a reduction in the levels of oxidative stress markers like MDA, GPx, and SOD measured in both striatum and hippocampus tissues [85]. In another study on the same PD rat models, pretreated with ellagic acid (50 mg/kg/day for one week), it was demonstrated that the supplementation reduced oxidative stress and apoptosis phenomena and improved MAO-B, nuclear factor (erythroid-derived 2)-like 2 (Nrf2), and heme oxygenase 1 (HO-1) [86]. Nrf2 and HO-1 are two critical elements of antioxidant mechanisms. Furthermore, a reduction in the loss of TH-positive neurons within the substantia nigra pars compacta was observed in these models. The loss of TH-positive neurons is responsible for reduced dopamine synthesis in the nigrostriatal system [86].

Finally, in recent work, the efficacy of urolithin A in preventing neurotoxicity was evaluated in cell cultures treated with 6-OHDA and in mouse models of PD. Urolithin A proved capable of inducing a reduction in cytotoxicity and apoptosis in vitro studies, while it improved motor deficits in animal models. Additionally, reduced neurotoxicity in nigral-striatal dopaminergic neurons and 6-OHDA-induced mitochondrial dysfunction were demonstrated [87].

Table 2 summarizes the characteristics of the studies regarding the effects of pomegranate or its extracts on PD.

### 3.2. Pomegranate and Skin

Among the various targets of aging, the skin, as the outermost organ with the function of a barrier against external hazards, is highly vulnerable to environmental damage [88,89]. In the epidermis, it leads to atrophy with slow cellular turnover and a decline in barrier function, resulting in flattening and thinning. In the dermis, collagen bundles produced by dermal fibroblasts are the main components that undergo significant changes such as fragmentation and disorganization, resulting in increased fragility, impaired vascular support, poor wound healing, reduced elasticity, and firmness, as well as the appearance of wrinkles and fine lines [90]. Over time, continuous exposure to environmental factors and intrinsic aging-related changes contribute to this chronic inflammation. The repercussions of skin inflammaging are also reflected systemically, with the production of pro-inflammatory mediators, as seen in conditions like AD and psoriasis [91,92,93]. In human fibroblasts stimulated in vivo with cytomegalovirus (CMV) and/or lipopolysaccharide (LPS), Wolf et al. demonstrated that cells obtained from elderly patients produced higher levels of pro-inflammatory cytokines, such as IL-6 and IL-8, compared to those from young patients, proving that chronological aging itself is a significant factor in skin inflammaging [94].

Since ancient times, preparations from various herbal species have been used as medicinal compounds to treat different conditions, including age-related problems [95,96]. Owing to the bioactive compounds present in the extracts, such as polyphenols, terpenoids, and flavonoids, interest in medicinal plants is rapidly growing. Researchers aim to better characterize the effects of these compounds to improve patients’ quality of life. This is especially crucial when discussing skin aging, as it significantly impacts social relationships, as well as psychological and emotional well-being. Previous reports on plant extracts have demonstrated their efficacy in treating skin diseases [97,98,99].

The following subsections will explore these aspects, providing insights into the mechanisms related to skin inflammaging and the protective effects of pomegranate compounds.

#### 3.2.1. Skin-Inflammaging Mechanism

Both the epidermis and dermis are affected by inflammaging. In the upper layer of the skin, keratinocytes play a crucial role in maintaining skin homeostasis, although the mechanisms related to aging remain largely elusive. Stem cells in the deeper layers of the epidermis enable its renewal and proper functioning. During the aging process, these cell niches decline, leading to a reduction in cellular turnover and, consequently, decreased skin protection [90]. Collagen 17A1 (COL17A1), a transmembrane structural component of the basement membrane, plays a fundamental role in maintaining the proper function of the stem cell niches. It is recognized as a protective factor in chronologically aged, photoaged, and acutely UV-irradiated human skin in vivo [100,101]. The loss of adhesion of stem cells to their niches leads to reduced rates of keratinocyte renewal and thinning of the epidermis [102]. Lower levels of COL17A1 are associated with reduced hair-follicle stem cells (HFSCs) and melanocyte stem cells (McSCs) in mouse models, acting through the Piezo1-calcium-TNF-α signaling pathway [103,104,105]. The exact mechanism of COL17A1 reduction is not fully understood, but metalloproteinases (MMPs) are believed to play a significant role. Increased levels of MMPs are associated with intrinsic and extrinsic aging-related changes [106,107]. This central role is more prominent in the dermis, where the abundant collagen ECM produced by fibroblasts can be specifically broken down by MMPs, interfering with wound healing and the production of age-related pro-inflammatory mediators such as IL-1β, IL-6, and IL-8. Another crucial factor is Transforming Growth Factor-β (TGF-β), which helps maintain the balance of ECM proteins by regulating collagen synthesis, deposition, and degradation [108]. A reduction in the TGF-β signaling pathway is associated with aging, and ROS play a pivotal role by reducing the expression of TβRII, which leads to decreased binding of TGF-β to the surface of dermal fibroblasts [109].

Another mechanism through which inflammaging can be triggered is the massive presence of free radicals, particularly ROS, which play a key role in damaging biological molecules such as lipids, DNA, and proteins [110]. The majority of intracellular ROS, such as hydroxyl (OH•), superoxide (O_2_^−^), nitric oxide (NO•), Peroxynitrite (ONOO^−^), hydrogen peroxide (H_2_O_2_), singlet oxygen (1 O_2_), and ozone (O_3_), are derived from mitochondria, but for aging-related conditions, the non-mitochondrial source plays a major role. An example of the latter is the Fanton reaction, in which bivalent iron ions catalyze H_2_O degradation with the production of OH•, which can also damage DNA molecules. The reduction of endogenous antioxidant factors or the overproduction of ROS due to exogenous influences causes an oxidative imbalance in cellular homeostasis, leading to the expression of various genes, including growth factors, inflammatory cytokines, and cell cycle regulatory molecules [111]. Excessive ROS production can compromise skin structure, resulting in the loss of cellular functions and ultimately causing cell death. Damaged keratinocytes are closely linked to the aging process and various age-related skin symptoms, such as dehydration, irritation, and wrinkle formation [112,113].

UV radiation is a major source of ROS, produced either by the direct absorption of incident rays by the cell and its components or through a photosensitization mechanism [114]. This ROS production, primarily driven by UVA in the range of 320–400 nm, leads to the activation of various signaling pathways, mainly MAPKs, which in turn activate the NF-κB pathway and the transcription of pro-inflammatory cytokines [115,116]. Also, UVB contributes to this damage by activating ERK1/2 and p38 signaling in epidermal UVB-exposed keratinocytes via ROS generation [117]. UVA-induced skin inflammation occurs through ROS-dependent activation of NF-κB, facilitated by the degradation of its regulatory protein IkBa and the release of free iron, which acts as an IkBa-independent activator of NF-κB [118]. The damaging effects are also reported in fibroblasts, where the activation of JNK and p38 kinase pathways, along with the release of labile iron, are involved in the activation of NF-κB [119]. In addition to ROS production, UV radiation induces mutations in the p53 tumor suppressor gene, which can lead to skin cancer [120,121].

All these mechanisms, therefore, play key roles in affecting the complex framework of skin homeostasis and aging-related problems [122,123]. Therefore, influences on inflammatory dysregulation patterns, as well as oxidative stress involvement, have been described in senescent cells [123,124].

#### 3.2.2. Anti-Inflammaging Skin Effects of Pomegranate Extract

As described, the concept of inflammaging includes several hallmarks that act at various levels. Inflammation, alteration of repair mechanisms, UV-induced ROS, and oxidative stress are the main pathways through which inflammaging operates, interacting and amplifying each other, creating a vicious cycle that significantly contributes to this deleterious process [125].

Pomegranate contains various active compounds present at different concentrations in the juice, peel, seed, flower, and oil, each with distinct properties and uses. The compounds in pomegranate extract have been reported to have greater beneficial properties compared to anti-inflammatory compounds such as red wine, green tea, apples, and vitamins E and C [126].

Various triggers can elicit skin inflammation, and pomegranate-derived compounds have demonstrated efficacy against these hazardous factors from different perspectives.

Huang et al. described the utility of punicalagin in regulating inflammatory responses in tumor necrosis factor-alpha (TNF-α) and interferon-gamma (IFN-γ)-stimulated keratinocytes. They showed suppression of the NF-κB pathway by interrupting the SIRT1/STAT3 axis and stimulation of the anti-inflammatory Nrf2/HO-1 signaling pathway. Moreover, punicalagin treatment reduced the production of pro-inflammatory cytokines such as IL-8, MCP-1, CCL5, CCL17, and CCL20 [127]. Similarly, Rapa et al. reported reduced nuclear translocation of p65-NF-κB and decreased release of TNF-α, IL-6, and IL-1β, along with improved Nrf2-related antioxidant enzymes in 5-fluorouracil (5FU)-induced inflammation in human keratinocytes. Additionally, treatment with pomegranate extract significantly inhibited apoptosis, reduced ROS generation, and promoted wound repair in 5FU-treated cells [128]. A dose- and time-dependent correlation in inhibiting pro-inflammatory pathways involving the NF-κB and MAPK cascade, including ERK1/2, JNK1/2, and p38 protein, was also demonstrated by Afaq et al. in their in vivo study. Using pomegranate fruit extract on human keratinocytes treated with UVB radiation, the authors described a significant reduction in the activation of these inflammatory pathways, as well as a decrease in the expression of cytokines such as IL-1, IL-6, and IL-8, which recruit inflammatory cells [129].

The effects of pomegranate extract on the arachidonic acid pathway, an important pro-inflammatory hub, have also been investigated by various authors [127,130,131,132,133]. Huang et al. reported downregulation of COX-2 and iNOS activity in human keratinocytes using a punicalagin-rich extract [127]. Similarly, an ex vivo animal study showed that topical application of pomegranate rind extracts downregulated COX-2 expression more effectively than treatment with tannins alone, indicating that tannins may not be entirely responsible for the anti-inflammatory activity of pomegranate [130]. In formaldehyde- and phenobarbital-wounded animal models, Hamouda et al. confirmed these findings, showing that treatment with pomegranate seed oil reduced COX-2 activity [131]. Finally, Khan et al. reported that oral administration of fresh pomegranate extract inhibited COX-2, iNOS, and matrix metalloproteinases-2, -3, and -9 expression in UVB-exposed mouse skin [132]. Conversely, in LPS-stimulated murine macrophages, the expression or activity of COX-2 was not affected by ellagic acid, gallic acid, and punicalagin extract from whole pomegranate. However, anti-inflammatory effects were reported through the reduction of NO, PGE-2, and IL-6 production [133]. Similar results were described in a murine model exposed to UVB radiation after topical application of the pomegranate active compound ellagic acid, with a reduction in IL-1β and IL-6 production and macrophage infiltration. The same paper also evaluated the effects on human fibroblasts and keratinocytes, showing reduced MMP-1 mRNA expression and ICAM-1 expression, respectively [134].

In literature, various researchers have evaluated the effects of pomegranate extract, alone or in combination with other substances, on cellular oxidative stress and ECM damage in vivo.

In human keratinocytes, the protective properties of a standardized commercial pomegranate extract and its phenolics, including punicalagin, ellagic acid, and urolithin A, were reported against hydrogen peroxide (H_2_O_2_)-induced oxidative damage. This study highlighted reduced ROS production, H_2_O_2_-stimulated caspase-3 activity, and apoptotic cell population in treated cells [135]. In the same model stimulated with PM-10, treatment with punicalagin and (–)-epigallocatechin-3-gallate (EGCG) decreased ROS production and, in a dose-dependent manner, inhibited the expression of NADPH oxidases (NOX)-1, NOX-2, and MMP-1, which are associated with oxidative stress and ECM degradation. Moreover, an anti-inflammatory effect was observed with reduced levels of TNF-α, IL-1β, IL-6, and IL-8 [136]. The same antioxidative activity, with a reduction of ROS, was reported by Quiles et al., using a combination of polyphenolic plant extracts (*Centella asiatica*, Pomegranate, *Citrus aurantium* var. *sinensis*, and Herba Cistanche) in human fibroblasts stimulated with H_2_O_2_ and UVB [137].

Another formulation containing *Ginkgo biloba*, pomegranate, Ficus carica, and Morus alba showed antioxidant effects in vivo. Ghimeray et al. demonstrated that the formulation inhibited collagenase in a dose-dependent manner, likely due to the synergistic effect of polyphenols causing conformational changes in the enzyme. Concerning the impact of oxidative stress on skin appearance, the in vivo part of this study reported reduced wrinkle depth in 21 women who used a 2% cream formulation for 56 days [138]. Furthermore, another in vivo study found that a pomegranate extract drink notably enhanced skin moisture, brightness, elasticity, and collagen density in female subjects after 8 weeks of treatment; moreover, high scavenging activity was reported in vivo [139]. Similarly, semi-purified anthocyanins from fresh pomegranate arils yielded comparable outcomes, with marked improvements in skin shape, hydration, wrinkle reduction, and firmness observed in twelve patients [140].

When discussing skin aging, the primary external factor that leads to the production of significant amounts of ROS and thus enhances inflammaging is UV exposure, known as photo-aging.

In UV-B (60 mJ/cm^2^) exposed human fibroblasts, Mariné-Casadó et al. reported a significant reduction in ROS levels using whole pomegranate extract, both in preventive and regenerative treatments. Additionally, they observed a substantial increase in pro-collagen type I, total collagen, and hyaluronic acid levels, along with a decrease in MMP-1 levels [141]. Similarly, Park et al. demonstrated that whole pomegranate-fruit extract inhibited MMP-1 expression and increased pro-collagen type I levels in UV-B (170 mJ/cm²) exposed fibroblasts [142]. Ellagic acid extracted from pomegranate, encapsulated into chitosan-coated niosomes, showed increased expression of genes implicated in collagen production (Co1A1) and downregulation of the main proteinase responsible for its degradation (MMP-3) in UV-A (wavelength λ of 365) exposed human fibroblasts [143]. Finally, Pacheco-Palencia et al. investigated the effects of a pomegranate fruit extract standardized to punicalagin on the same cell type. Their findings included a reduction in ROS generation, cell death, caspase-3 expression, and pro-inflammatory NF-κB activation in treated UVA- and UVB-exposed (60 mJ) human fibroblasts [144].

In UVB-exposed keratinocytes, pre-treatment with polyphenol-rich pomegranate fruit extract reduced the upregulation of MMP-1, -2, -7, and -9, as well as the phosphorylation of MAPKs and c-jun induced by UVB (15–30 mJ/cm^2^) [145]. Other researchers focused on UVB-mediated DNA damage (90 or 200 mJ/cm^2^), reporting a dose-dependent reduction in these harmful effects when the cells were pre-treated with a pomegranate seed oil nano-emulsion [146].

In murine UVB-irradiated models (0.18 J/cm^2^), Kang et al. reported an increase in skin water content, collagen type I, and hyaluronan levels using an oral dose of pomegranate extract. IL-1β levels and MPO activity were lower, and IL-10 was higher in the treated model compared to the control group. Moreover, reduced expression of skin MMP-1, -9, and -13, as well as Nox2 mRNA, was reported. Histopathological findings highlighted significant dose-dependent decreases in dermis sclerosis and inflammatory signs in treated mice [147].

Finally, the skin-microbiota changes with pomegranate extract and juice treatment were analyzed in 74 female patients after UVB exposure (220–550 mJ/cm^2^). The authors did not report significant changes in skin microbiota composition, but treatment led to an increased minimum erythema dose in treated patients compared to the control group. However, this study did not clarify the correlation between these changes and photoprotection [148].

Since the skin is one of the tissues most susceptible to continuous lesions, wound-healing processes have been the focus of numerous studies. In fact, these processes are significantly affected by aging, as elderly skin exhibits a reduced ability to heal.

There is evidence of the effects of pomegranate extracts on the regeneration processes of damaged skin. The first study using mouse models dates back to 2004. Murthy et al. [149] evaluated wound healing activity by studying skin contraction, hydroxyproline collagen content, and the histopathological evaluation of the tissue. Wound healing times were significantly reduced in mice treated with 5% pomegranate peel extract gels (10 days) compared to mice treated with 2.5% gels (12 days) and controls (16–18 days). Furthermore, the hydroxyproline content was doubled in mice treated with the 5% gel compared to the control group. From then until today, several in vivo studies on animal models have demonstrated the effectiveness of pomegranate extracts in inducing wound healing quickly [150,151,152,153,154,155]. Yuniarti et al. [156] tried to use pomegranate extract standardized to 40% ellagic acid, demonstrating the superiority of the 7.5% extract in reducing collagen deposition, neutrophil infiltration, angiogenesis, and degree of fibrosis. In 2006, Aslam et al. studied, for the first time, the mechanisms through which pomegranate extracts can enhance the skin’s healing process [157]. The in vivo studies showed pomegranate seed oil’s proliferation-stimulating effect without affecting fibroblast function. Peel extracts, instead, promote the synthesis of type I pro-collagen and inhibit the production of MMP-1 by dermal fibroblasts [157]. In vivo studies on animal models have demonstrated the effects of pomegranate extracts on the production of VEGF-A and TGF-β1. The immunohistochemical study of second-degree burns treated with these extracts demonstrated an increase in VEGF-A and TGF-β1 after seven days of treatment, maintaining high levels until day 14 and decreasing after 21 days. In contrast, VEGF-A and TGF-β1 levels in the model group increased after 28 days [158].

Table 3 resumes the characteristics of the reported studies about the beneficial effects of pomegranate on skin inflammaging.

### 3.3. Pomegranate in Other Diseases

Studies regarding the use of pomegranate extracts to treat other pathologies are limited in the literature.

The phenomenon of inflammaging also involves the cardiovascular system. The dynamic interplay between pro-inflammatory cytokines, inflammasomes, senescent cells, and age-related immune changes creates and maintains chronic low-grade inflammation, which increases the risk of developing cardiovascular diseases [159]. In 2016, Dos Santos et al. [160] studied the influence of pomegranate consumption on systolic blood pressure and coronary angiotensin-converting enzyme (ACE) activity. They used mouse models of arterial hypertension, treating them for 30 days with pomegranate extract or filtered water. Pomegranate extracts significantly reduced systolic blood pressure and coronary ACE activity. In the animals subjected to treatment, significantly lower superoxide anion levels were observed, while in the untreated group, coronary morphology demonstrated a total increase in vascular wall areas. Subsequent supplementation in the control group decreased this effect, demonstrating how the consumption of pomegranate peel extract can provide protection against hypertension [160]. A recent study demonstrated the efficacy of extracts derived from pomegranate-derived bioactive and non-edible compounds (PPE) in animal models of hypertension. PPE, administered for six weeks, demonstrated efficacy in maintaining systolic blood pressure compared to the reference drug (Captopril). Furthermore, PPE has anti-inflammatory and antifibrotic effects [159].

In Unani and Ayurvedic medicine, pomegranate flower extract is used to treat diabetes. Wang et al. [161] conducted a 55-week study on mice receiving pomegranate flower powder supplementation. The treatment proved effective in improving aging-induced insulin resistance.

Some authors wanted to study the effects of supplementation with pomegranate extracts in counteracting the inflammation and aging of the liver [162]. It has been demonstrated that at the liver level, aging causes excessive apoptosis of hepatocytes and, in some cases, liver fibrosis. Furthermore, aging can cause dysfunction in some proteins, such as insulin-like growth factor 1 (IGF-1), telomerase reverse transcriptase (TERT), and cytochrome P450. Alshinnawy et al. [162] evaluated the efficacy of Ta-65, a potent telomerase activator, administered in combination with pomegranate extracts to reduce age-induced liver deterioration. The combined administration to aged rats normalized serum levels of total proteins, globulins, and IGF-1. The antioxidant activity of pomegranate also restored the cellular antioxidant environment and reduced hepatic MDA and protein carbonyls.

The same therapeutic combination was used to treat infertility and renal dysfunction in male mouse models [163]. Aging causes a hormonal imbalance, resulting in sperm abnormalities and a reduction in the number and motility of sperm. Furthermore, increased levels of serum creatinine, uric acid, sodium, and potassium are found. Treatment with Ta-65 or pomegranate has proven effective in restoring age-induced changes and counteracting male infertility [163]

## 4. Discussion

Pomegranate is a fruit rich in biologically active compounds with numerous health benefits. Numerous studies in the literature have shown the beneficial effects of pomegranate extracts on inflammation and oxidative stress, as well as on slowing down the mechanisms of aging. The ensemble of these mechanisms represents inflammation, and studies focus mainly on the central nervous system and the skin.

The neuroprotective effects of pomegranate and its compounds have been studied for several years, and the research has mainly focused on cellular or animal models of AD [41]. The mechanisms of action are different. Oral supplementation with pomegranate or its extracts reduces oxidative stress and rebalances the activity of the main antioxidant enzymes [164]. The anti-inflammatory action is notable: reduced pro-inflammatory cytokines (IL-1β, IL-6, and TNF-α), inhibition of eotaxin and NF-κB transcription, and a dose-dependent reduction in NO and PGE2 production [50,62]. Pomegranate also has anti-amyloidogenic effects, helping to reduce the formation and deposition of Aβ plaques [49].

The most active compounds in this sense are punicalagin and ellagic acid [63,64]. Urolithins, resulting from the intestinal metabolism of polyphenols and ellagitannins, are an essential inducer of mitophagy and are important for counteracting oxidative stress and damage induced by free radicals in the CNS [71]. Therefore, the importance of the intestinal microbiota and the close connection between the intestine and the CNS are evident. Few studies regarding PD are in the literature. However, these studies are primarily in vivo. Supplementation with pomegranate extracts has good efficacy in slowing down the depletion of dopaminergic neurons and improving both pyramidal and extrapyramidal symptoms in treated mouse models. Furthermore, pomegranate exerts its neuroprotective effects by inhibiting apoptosis, reducing inflammation and oxidative stress, and promoting the release of anti-inflammatory cytokines such as IL-10 [83]. Despite these results, a single study reported the poor efficacy of supplementary treatment with pomegranate extracts, the worsening of manifestations, and increased disease biomarkers [81].

Pomegranate also exerts its benefits at the skin barrier level. Extracts applied, especially in topical formulations, have often been reported to have encouraging results in counteracting the mechanisms of skin inflammation, both in vivo and in vivo studies.

Pomegranate extracts exert a potent anti-inflammatory action by suppressing the SIRT1/STAT3 axis and the NF-κB pathway, as well as the production of pro-inflammatory cytokines such as IL-1, IL-6, and IL-8 [127]. Another anti-inflammatory mechanism that has come to light concerns the reduction of the expression of COX-2 and iNOS [127]. Above all, polyphenols have proven helpful in inhibiting the activity of metalloproteinases and collagenases, reducing the degradation of the extracellular matrix [142,145]. These compounds have also found utility, in the form of oils or emulsions, in preventing UVB-induced inflammation and skin aging. Finally, considering that skin regeneration is a physiological mechanism strongly influenced by age and the inflammatory microenvironment, gel extracts based on pomegranate promise great effectiveness in reducing healing times.

Despite this extensive evidence, there are no ongoing clinical trials regarding the supplementation of pomegranate or its metabolites for the treatment of patients with neurodegenerative diseases. On the contrary, several topical preparations on the market contain extracts of the fruit that are used for their anti-aging and anti-inflammatory skin effects, but even in this case, clinical trials for skin pathological conditions are lacking.

The lack of human studies is not justified by the potential side effects that pomegranate supplementation or its metabolites could determine. There is no evidence of side effects or toxicity induced by pomegranate.

Studies concerning other organs or systems are limited. Supplementation with pomegranate extracts may benefit in preventing cardiovascular diseases involving pathogenetic mechanisms underlying chronic low-grade inflammation and aging, such as arterial hypertension and atherosclerosis [159,160]. Application margins could also concern age-induced liver damage [162], insulin resistance [161], and age-induced infertility [163].

## 5. Conclusions

Inflammaging is undoubtedly a new and complex concept resulting from the union of different mechanisms, including aging, oxidative stress, and chronic low-grade inflammation. The literature provides various evidence regarding the ability of pomegranate extracts to counteract these mechanisms. However, there are some limitations. The studies mainly concern neurodegenerative and skin diseases, while studies concerning other fields of application need to be more practical. Furthermore, no human studies demonstrate this fruit’s anti-inflammaging effects. Despite this, the phytotherapeutic potential of this fruit and its extracts is notable, and their therapeutic properties vary. Considering the evidence reported and the practically absent side effects that could accompany supplementation with pomegranate, it would be helpful to start considering the use of these compounds for treating neurodegenerative and skin diseases in humans. In the future, supplementation with pomegranate extracts, polyphenols, or urolithins could represent a valuable low-risk complementary therapy for patients with difficult-to-manage diseases such as AD or PD but also a valid therapeutic alternative for topical or systemic treatment of skin pathologies.

## Figures and Tables

**Table 1:** Main studies and their characteristics found through our research on the effects of pomegranate extract in AD.

Author	Study Model	Aims	Outcome
Choi et al. [48]	In vitro (PC12 cells) and in vivo (mice)	To study the antioxidant and neuroprotective effects of pomegranate against oxidative stress-induced cytotoxicity.	Pomegranate ethanolic extracts protected PC12 cells from H_2_O_2_-induced oxidative stress. Furthermore, in mouse models, pomegranate inhibited neuronal cell death caused by Aβ-induced oxidative stress and Aβ-induced learning and memory deficits.
Velagapudi et al. [49]	In vitro (SK-N-SH cells)	To evaluate the effects of freeze-dried pomegranate on PGE2 production in IL-1β-stimulated SK-N-SH cells.	There was a dose-dependent reduction in COX-2-dependent PGE2 production and an inhibitory effect on NF-κB transactivation and BACE-1 expression.
Essa et al. [50]	In vivo (APPsw/Tg2576 mice)	To investigate the beneficial effects of dietary supplements such as pomegranate, figs, or dates on suppressing inflammatory cytokines.	Supplementation significantly reduced levels of inflammatory cytokines (IL-1β, IL-2, IL-3, IL-4, IL-5, IL-6, IL-9, IL-10, and TNF-α) and the activity of eotaxin, while also delaying the formation of senile plaques.
Kumar et al. [51]	In vivo (scopolamine treated mice)	To evaluate the effect of ethanolic extract of pomegranate seeds on the cognitive performance of aged and scopolamine-treated young mice.	Chronic administration of Pomegranate and vitamin C significantly reversed age- or scopolamine-induced retention deficits, reduced the level of lipid peroxidation, and increased the level of antioxidant glutathione in brain tissues.
Subash et al. [52]	In vivo (APPsw/Tg2576 mice)	To evaluate the antioxidant effects of polyphenols on animal models	Supplementation with 4% pomegranate attenuated oxidative damage by reducing LPO and protein carbonyl levels and by restoring the activity of antioxidant enzymes.
Ahmed et al. [53]	In vivo (R1.40 transgenic mice)	To demonstrate the anti-AD effects of pomegranate extract	The extract did not demonstrated benefits on cognitive performance but demonstrated an anti-amyloidogenic effect. This benefit may be related to effects on the γ-secretase enzyme.
Braidy et al. [54]	In vivo (APPsw/Tg2576 mice)	To study the effects of dietary supplementation of 4% pomegranate extract on neuroinflammation and synaptic plasticity	Supplementation for 15 months reduced the loss of synaptic structure proteins (PSD-95, Munc18-1, SNAP25, and synaptophysin), the phosphorylation of p-CaMKIIα/CaMKIIα and pCREB/CREB, and neuroinflammatory activity. Furthermore, enhanced autophagy and activation of the mammalian target of rapamycin signaling pathway were observed.
Morzelle et al. [55]	In vivo (C57Bl/6 mice)	To demonstrate the effects of pomegranate peel extract on spatial memory, neuroplasticity biomarkers, oxidative stress, and inflammation	Consumption of pomegranate reduced amyloid plaque density, increased the expression of the neurotrophin BDNF, and reduced the activity of the enzyme acetylcholinesterase without side effects.
Almuhayawi et al. [56]	In vivo (AlCl 3-induced AD rat model)	To evaluate the therapeutic and protective effects of pomegranate extract in standard formulation and in nanoparticles	Pomegranate extract has proven effective in preventing oxidative damage and reducing histopathological signs of AD. Extract-loaded nanoparticles demonstrated greater efficacy.
Khokar et al. [57]	In vitro	To determine the phenolic content of Omani pomegranate peel extracts and study their antioxidant and anticholinesterase activities.	Butanol extract is rich in phenolic compounds and has excellent antioxidant activity, demonstrating anti-AD effects.
Karagecili et al. [58]	In vitro	To evaluate the antioxidant effects of polyphenols.	The ethanolic extract of pomegranate was found to be rich in phenolic content, exhibiting antioxidant effects and reducing power and the ability to inhibit AChE, α-glycosidase, α-amylase, and hCA II.
Ali et al. [59]	In vitro (Aβ42-induced N2a/APP cells)	To evaluate the anti-AD effects of pomegranate extracts and which of these is the most active	Among the various compounds, gallagic acid and castalagin markedly reduced the secretion of Aβ peptides, the production of ROS, and the expression levels of BACE1 and APPsβ.
Rojanathammanee et al. [60]	In vitro (Primary microglia culture) + in vivo (APP/PS1 transgenic mice)	Evaluation of the effects of pomegranate extract supplementation (6.25 mL/L) over three months in attenuating microgliosis.	The treatment improved the mice’s mnemonic functions, reducing the concentration of TNF-α and the transcriptional activity of NFAT. In vitro studies demonstrated that punicalagin and ellagic acid attenuated NFAT activity and decreased TNF-α secretion.
Kim et al. [62]	In vitro (astrocytes and microglial BV-2 cells)	To investigate the effects of punicalagin on memory deficiency induced by LPS	Punicalagin demonstrated neuroprotective effects by inhibiting LPS-induced expression of iNOS and Cox-2 and the production of ROS, NO, TNF-α, and IL-1β. It also inhibited LPS-induced NF-κB activation and Aβ1–42 generation.
Clementi et al. [64]	In vitro (human neuroblastoma IMR-32 cells)	To evaluate the effects of resveratrol and punicalagin on the enzymatic activity of methionine sulfoxide reductase A (MsrA)	Pretreatment with resveratrol and punicalagin increased the expression and enzymatic activity of MsrA, resulting in a lowering of the oxidative potential of the cells and a protective effect on the CNS.
Chen et al. [63]	In vivo (D-gal-induced brain aging mouse model)	To evaluate the neuroprotective effects of punicalagin in animal models	Punicalagin improved learning and memory deficits by reducing neuroinflammation by inhibiting microglial activity. In mouse models, a reduction in MDA and ROS and an inhibition of the NLRP3 inflammasome were observed.
Yuan et al. [71]	In vitro + in vivo (*Caenorhabditis elegans*).	To demonstrate which of the main compounds has the characteristics to pass the blood–brain barrier and determine its anti-AD properties	Among the 21 components studied, only urolithins have been shown to have the full characteristics to pass the blood–brain barrier. Urolithins prevented β-amyloid fibrillation in vitro, and methyl-urolithin B demonstrated a protective effect in animal models.
DaSilva et al. [73]	In vitro (murine BV-2 microglia, human SH-SY5Y neurons)	To evaluate the effects of urolithins on neuroinflammation and search for biomarkers	Urolithins reduce nitric oxide, IL-6, PGE2, and TNF-α levels in microglia. Urolithins also reduce apoptosis and the release of caspase 3/7 and 9 caused by H_2_O_2_-induced oxidative stress.

**Table 2:** Main studies and their characteristics found through our research on the effects of pomegranate extract in PD.

Author	Study Model	Aims	Outcome
Braidy et al. [80]	In vitro (human primary neuronal cells)	To evaluate the neuroprotective effects of pomegranate juice extracts against MPTP-induced neurotoxicity.	Pomegranate juice can reverse the effects of MPTP on antioxidant enzyme activities and attenuate neurotoxicity in a dose-dependent manner. The Helow and Malasi varieties have proven superior to the Qusum and Hamadh.
Tapias et al. [81]	In vivo (rotenone-induced parkinsonism mouse models)	To evaluate the protective effects against PD of pomegranate juice.	Pomegranate juice did not mitigate the disease, but increased nigrostriatal terminal depletion, loss of dopaminergic neurons, inflammatory response, and caspase activation, thus increasing neurodegeneration.
Małgorzata Kujawska et al. [82]	In vivo (rotenone-induced parkinsonism mouse models)	To evaluate the protective effects against PD of pomegranate juice	Pomegranate juice improved postural stability and neuronal survival and protected against oxidative damage. Furthermore, supplementation reduced α-synuclein aggregation and increased mitochondrial aldehyde dehydrogenase activity, maintaining the antiapoptotic protein Bcl-xL at the control level.
Małgorzata Kujawska et al. [83]	In vivo (rotenone-induced parkinsonism mouse models)	To study the ability of pomegranate juice to protect against olfactory, motor, and neurochemical alterations in PD.	Pomegranate juice treatment protected against rotenone-induced depletion of dopaminergic neurons in the midbrain, resulting in improved olfactory function and vertical activity.
Fathy et al. [84]	In vivo (Paraquat-induced parkinsonism mouse models)	To evaluate the protective effects of pomegranate seed extract and juice against PD.	The treatment led to an increased level of tyrosine hydroxylase, dopamine, and its metabolite in the striatum, also improving oxidative stress and significantly inhibiting the expression of the striatal NF-κB gene. A reduction in apoptosis and a decrease in pro-inflammatory cytokines were observed, with a significant increase in IL-10 levels.
Sarkaki et al. [85]	In vivo (6-OHDA lesioned mice)	To study the effects of ellagic acid on motor disorders, local pallidal EEG, and its frequency band power, and brain antioxidant content.	Ellagic acid improves motor deficits and electrophysiological performance by increasing brain antioxidant content.
Baluchnejadmojarad et al. [86]	In vivo (6-OHDA lesioned mice)	To study the effects of ellagic acid.	Ellagic acid attenuated rotational distortion, reduced onset latency and total time in the narrow beam task, decreased striatal MDA levels, ROS, and DNA fragmentation, and enhanced antioxidant expression.
Liu et al. [87]	In vitro (PC12 cells) + In vivo (6-OHDA lesioned mice)	To understand the mechanisms underlying the role of urolithin A in 6-OHDA-induced neurotoxicity.	Urolithin A was effective in inducing protection against 6-OHDA-induced cytotoxicity and apoptosis in cellular models. Furthermore, administration to mouse models improved both motor deficits and nigral-striatal dopaminergic neurotoxicity, while attenuating mitochondrial dysfunction.

**Table 3:** Main studies and their characteristics found through our research on the effects of pomegranate extract on skin-inflammaging.

Authors	Sample	Hazardous Factor	Pomegranate Formulation	Outcome	Activity
Huang et al. [127]	In vivo (Human keratinocytes)	Stimulation with TNF-α and IFN-γ	Punicalagin isolated from the peel	Suppression of the NF-κB pathway; stimulation of the Nrf2/HO-1 signaling pathway; reduction in IL-8, MCP-1, CCL5, CCL17, and CCL20; downregulation of COX-2 and iNOS activity.	Anti-inflammatory
Rapa et al. [128]	In vivo (Human keratinocytes)	Stimulation with 5-FU	Juice extract	Reduction in NF-κB pathway activation and the release of TNF-α, IL-6, and IL-1β; stimulation of Nrf2-related enzymes; reduced apoptosis; reduced ROS generation; improved wound repair.	Anti-inflammatory; Antioxidative stress; Pro-healing
Afaq et al. [129]	In vivo (Human keratinocytes)	UVB	Whole fruit extract	Reduction in NF-κB and MAPK inflammatory pathway activation and the production of IL-1, IL-6, and IL-8.	Anti-inflammatory; photo-aging protection
Houston et al. [130]	Ex vivo (Full thickness porcine skin)	None	Rind extract (topical)	Downregulation of COX-2.	Anti-inflammatory
Hamouda et al. [131]	In vivo (Mice)	Phenobarbital and formaldeyde	Seed oil extract (topical)	Downregulation of COX-2.	Anti-inflammatory
Khan et al. [132]	In vivo (Mice)	UVB	Whole fruit extract (oral)	Downregulation of COX-2, iNOS, and MMP-2, -3, and -9.	Anti-inflammatory; photo-aging protection
BenSaad et al. [133]	In vivo (Murine macrophages)	LPS	Ellagic acid, gallic acid, and punicalagin	Reduction in NO, PGE-2, and IL-6 production.	Anti-inflammatory
Bae et al. [134]	In vivo (Human fibroblasts and keratinocytes) + In vivo (mice)	UVB	Ellagic acid (topical in mice)	Reduction in IL-1b and IL-6 and macrophages infiltration in mouse skin sample; reduction in MMP-1 mRNA expression in human fibroblasts and ICAM-1 expression in human keratinocytes.	Anti-inflammatory
Liu et al. [135]	In vivo (Human keratinocytes)	H_2_O_2_	Fruit extract, punicalagin, ellagic acid, and urolithin A	Reduction in ROS production and H_2_O_2_-stimulated caspase-3 activity.	Antioxidative stress
Seok et al. [136]	In vivo (Human keratinocytes)	PM10	Punicalagin and (-)-epigallocatechin-3-gallate	Reduction in ROS production and the expression of NOX-1, NOX-2, TNF-α, IL-1β, IL-6, IL-8, and MMP-1.	Antioxidative stress; anti-inflammatory
Quiles et al. [137]	In vivo (Human fibroblast)	H_2_O_2_	Pomegranate, Centella asiatica, Citrus aurantium var. sinensis, and Herba Cistanche fruits extract	Reduction of ROS production	Antioxidative stress
Ghimeray et al. [138]	In vivo (21 female patients) + in vivo (spectrophotometric evaluation)	H_2_O_2_; O_2_^−^; 1,1-diphenyl-2-picrylhydrazyl (in vivo)	Pomegranate, Ginkgo biloba, Ficus carica, and Morus alba fruit extract (2% topical cream in vivo)	High scavenging activities and dose-dependent inhibition of collagenase (in vivo); reduced wrinkle depth (in vivo).	Antioxidative stress; anti-aging
Chan et al. [139]	In vivo (80 female patients) + in vivo (spectrophotometric evaluation)	1,1-diphenyl-2-picrylhydrazyl; NO·	Gallic acid-rich fermented extract (oral)	High scavenging activities (in vivo); improvement in moisture, brightness, elasticity, and collagen density (in vivo).	Antioxidative stress; anti-aging
Abdellatif et al. [140]	In vivo (12 patients)	None	Anthocyanin from fresh arils extract (topical)	Improvements in skin shape, hydration, wrinkle reduction, and firmness	Anti-aging
Mariné-Casadó et al. [141]	In vivo (Human fibroblast)	UVB	Natural extract	Reduction in ROS levels; increase in pro-collagen type I, total collagen, and hyaluronic acid levels, along with a decrease in MMP-1 levels	Photo-aging protection
Park et al. [142]	In vivo (Human fibroblast)	UVB	Whole fruit extract	Reduction in MMP-1 expression and increased pro-collagen type I levels	Photo-aging protection
Abd-Elghany et al. [143]	In vivo (Human fibroblast)	UVA	Ellagic acid	Increased expression of the Co1A1 gene; downregulation of MMP-3.	Photo-aging protection
Pacheco-Palencia et al. [144]	In vivo (Human fibroblast)	UVA; UVB	Punicalagins-rich extract	Reduced transcription of NF-κB and ROS production; downregulation of caspase-3.	Anti-inflammatory; antioxidative stress; Photo-aging protection
Zaid et al. [145]	In vivo (Human keratinocytes)	UVB	Polyphenol-rich fruit extract	Reduced upregulation of MMP-1, -2, -7, and -9, and phosphorylation of MAPKs and c-jun	Anti-inflammatory; Photo-aging protection
Baccarin et al. [146]	In vivo (Human keratinocytes)	UVB	Seed oil nanoemulsion	Reduced cell DNA damage in a dose-dependent manner	Photo-aging protection
Kang et al. [147]	In vivo + in vivo + ex vivo (mice)	UVB	Juice concentrated powder (oral)	Reduction in skin wrinkles, improvement of skin water contents, collagen type I, and hyaluronan contents (in vivo); reduced IL-1β levels and MPO activity; increased IL-10 levels; reduced skin MMP-1, -9, and -13, and Nox2 mRNA expression (in vivo); dose-dependent decreases in dermis sclerosis and inflammation (ex vivo).	Anti-inflammatory; Photo-aging protection; Anti-aging
Henning et al. [148]	In vivo (74 female patiens)	UVB	Whole fruit extract and juice extract	No significant changes in the skin microbiota composition; increased of minimum erythema dose	Photo-aging protection
Murthy et al. [149]	In vivo + ex vivo (mice)	Wound	Peel extract (topical)	Reduction of wound healing (in vivo); increased hydroxyproline content (ex vivo).	Pro-healing
Hayouni et al. [150]	In vivo (pigs)	Wound	Peel extract (topical)	Increased wound contraction and decreased period of epithelialization, collagen, DNA, and proteins synthesis.	Pro-healing
Nayak et al. [151]	In vivo + ex vivo (mice)	Wound	Peel extract (topical)	Increased wound contraction, decreased period of epithelialization (in vivo); increased hydroxyproline content (ex vivo).	Pro-healing
Zekavat et al. [152]	In vivo (mice)	Wound	Peel extract (topical)	Reduced wound healing time.	Pro-healing
Nasiri et al. [153]	In vivo (mice)	Wound	Flower extract (topical)	Increased wound contraction.	Pro-healing
Elzayat et al. [154]	In vivo (mice)	Wound	Pomegranate, henna, and myrrh extract (topical)	Increased wound contraction and decreased period of epithelialization.	Pro-healing
Lukiswanto et al. [155]	Ex vivo (mice)	Wound	Ellagic acid rich-whole fruit extract	Decreased period of epithelialization; increased density of collagen and angiogenesis; reduced number of inflammatory cells (PMNs)	Pro-healing; anti-inflammatory
Yuniarti et al. [156]	Ex vivo (mice)	Wound	Ellagic acid rich-whole fruit extract	Increased density of collagen and angiogenesis; reduced number of inflammatory cells (PMNs).	Pro-healing; anti-inflammatory
Aslam et al. [157]	In vivo (Human keratinocytes and fibroblasts)	None	Peel, juice, and seed extract	Stimulation of keratinocytes proliferation (seed oil); increased type I procollagen synthesis and inhibition of MMP-1 production (peel extract).	Pro-healing; anti-inflammatory
Zhang et al. [158]	In vivo + ex vivo (pigs)	Wound	Peel extract	Reduction in epithelialization and fur growing time (in vivo); early VEGF-A and TGF-β1 mRNA expression.	Pro-healing

## Data Availability

No new data were created or analyzed in this study. Data sharing is not applicable to this article.
